# Acute and Sub-Chronic Toxicological Evaluation of *n*-Hexane Fraction of *Uvaria chamae* Leaves

**DOI:** 10.21010/Ajidv19i2S.11

**Published:** 2025-10-17

**Authors:** V.O Bamimore, E.D Manuel-Mosi, O.O Fayehun, J.I Olawuni, E Ogunwole, C.A Elusiyan, G Olayiwola

**Affiliations:** a Department of Pharmacognosy University of Medical Sciences, Ondo City, Ondo State, Nigeria; b Department of Physical and Chemical Sciences, Elizade University Ilara-Mokin, Ondo State, Nigeria; c Department of Integrated Science, Adeyemi Federal University of Education, Ondo State, Nigeria; d Department of Biochemistry and Molecular Biology, Obafemi Awolowo University, Ile-Ife, Osun State, Nigeria; e Department of Physiology, University of Medical Sciences, Ondo, Ondo State, Nigeria; f Department of Clinical Pharmacy and Pharmacy Administration, Obafemi Awolowo University, Ile-Ife, Osun State, Nigeria

**Keywords:** *Uvaria chamae*, *n-*hexane, acute toxicity

## Abstract

**Background:**

The plant species identified as *Uvaria chamae*, a member of Annonaceae has exhibited notable anti-trypanosomal effect in *in-vivo* studies, demonstrating potential therapeutic benefits in animal models. However, a comprehensive toxicity profile is essential to assess the safety of *U. chamae* for potential therapeutic applications. This study investigated the toxicity of *U. chamae* leaves (*n*-hexane fraction) in order to identify any potential adverse effects and establish a safer dosage threshold for prolonged use.

**Methods:**

The *n*-hexane partitioned fraction obtained from *U. chamae* leaves was subjected to acute and sub-chronic toxicity evaluations. The short-term toxicity assessment followed established procedures. For the sub-chronic study, a total of 30 animals received the fraction continuously for 35 days. Biochemical analyses were performed on serum and liver homogenates to assess key parameters, including Aspartate aminotransferase (AST) and Alanine aminotransferase (ALT), Triglycerides (TRIG) and Total cholesterol (TC). Also, histopathological examinations were performed on selected tissue samples.

**Results:**

The acute toxicity assessment showed an LD_50_ value exceeding 5000 mg/kg. For the repeated dose toxicity study, results showed statistically significant effect (p < 0.05) variations in AST and ALT levels within both liver and serum homogenates in relation to dosage. Additionally, histopathological analysis identified morphological alterations that distinct are from the control group and consistent with those typically observed in damaged tissues.

**Conclusion:**

At high doses, the n-hexane fraction of *U. chamae* showed no acute toxicity. However, prolonged use caused notable biochemical and morphological changes. A dose below 200 mg/kg is recommended for extended use.

## Introduction

Nature provides a wide array of bioactive compounds from which many important drugs have been obtained (Singh *et al.*, 2024). Among these natural remedies, *Uvaria chamae* is frequently used as a therapeutic agent in African herbal medicine. This study seeks to assess the potential toxicity of *U. chamae* leaves’ *n*-hexane fraction by conducting both acute and sub-chronic toxicity evaluations.

*Uvaria chamae* (P. Beauv), Annonaceae, is a climbing small tree or shrub, indigenous to the tropical woodlands of West and Central Africa (Okwu and Iroabuchi, 2004). Its most common names are finger root and bush banana (Omogbai and Eze, 2011). In Nigeria, it is known by various local names: “Ẹruju” or “Oko Ọja” in Yoruba dialect, “Kas Kaifi” in the north, “Mmimi Ohea” or “Nnuenwe” in Igboland, and “Awukolo” in Igala (Oliver, 2010). In Nigeria the root-bark prepared as decoction, is used for the treatment of vermifugal and stomachic conditions (Adams and Moss, 1999). Additionally, the sap extracted from its vegetative parts is applied in treating nosebleeds, open injuries, respiratory disorders, heart conditions, hematuria, hemorrhoids, and fever (Etukudo, 2003). The root decoction is also being taken orally and as a body-wash for oedematous condition (Olumese *et al.*, 2018). In Ghana, the root back decoction is used to combat dysentery (Marshall *et al.*, 2000). In Ivory Coast and Sierra Leone, it is often prepared with spices to treat jaundice and fevers (Burkill, 1994; Ayedoun *et al.*, 1999). In Senegal, the root decoction is used to treat infantile rickets while in Togo it is used to ameliorate pains during child-birth (Ijioma *et al.*, 2020). Equally in Senegal, the leaves and roots of *U. chamae* are macerated together and taken orally as cough remedy, being pulverized together with *Annona senegalensis* and to relieve renal-related issues (Burkill, 2004).

Pharmacologically, *Uvaria chamae* root ethanol extract demonstrated anti-inflammatory, antimicrobial, oxytocic, hepatoprotective, antivenom and blood sugar-lowering activities (Okwu and Iroabuchi, 2004; Soromou *et al.*, 2012; Emordi *et al.*, 2015). Additionally, the ethanol extract derived from its seeds has demonstrated antioxidant potential (Ita, 2017). Studies on different plant parts have revealed distinct bioactivities; for instance, the methanol extract from its leaves, the methanol fraction from its stem bark, and the ethanolic extract of the stem bark have exhibited cytotoxic effects (Philipov *et al.*, 2000; Awodiran *et al.*, 2018; Jalil *et al.*, 2020). Furthermore, three novel C-benzylated flavanones and several alkaloids, including chamuvarinin and nornantenine, have been identified as its bioactive constituents with cytotoxic properties (Philipov *et al.*, 2000; Derbré *et al.*, 2007).

Despite these vast medicinal usage of *U. chamae*, extensive studies to evaluate its toxicological information remain limited (Awaraka *et al.*, 2019). The *n*-hexane fraction was selected for evaluation due to its previously reported antitrypanosomal and anti-inflammatory activities raising the need for a thorough assessment of its safety (Adelodun *et al.*, 2013). This study investigated the acute and repeated dosing toxicity studies to provide vital information on its possible potential risks.

## Materials and Methods

### Experimental Animals

The study utilized animals procured from the experimental animal unit at the Faculty of Pharmacy, Obafemi Awolowo University (OAU), Ile-Ife, Nigeria. The experimental animals were housed in well-ventilated cages, provided with standard pelletized rodent feed and allowed unrestricted water supply. The handling and care of the experimental animals were carried out following the outlines of the National Research Council to guarantee animal welfare protocol (NRC, 2011). Swiss albino mice (*Mus musculus*, 19–25 g) and Swiss albino rats (*Rattus norvegicus domestica*, 180 – 230 g) of both sexes were utilized for the short and repeated dosing toxicity studies, respectively. The Ethical Clearance protocol for this research was submitted to the Ethics and Research Committee (ERC) of the Obafemi Awolowo University, and approved with Ethical Clearance Certificate No. ERC/2018/08/01

### Plant Collection and Preparation

Fresh leaves of *Uvaria chamae* were gathered from Obafemi Awolowo University, in Nigeria. A catalogue specimen (IFE 16791) was preserved for record keeping at the Ife Herbarium. The collected leaves were allowed to dry at room temperature and then ground using a laboratory blender. The powdered leaves were subjected to maceration in 70% methanol for 72 hours, with continuous stirring. After filtering the mixture through Absorbent cotton and laboratory filter paper, the derived solution was dried *in vacuo* under reduced pressure at 40°C via rotary evaporation. The resulting crude extract was freeze-dried, yielding a total of 7.3%. In the next step, 60 g of the obtained crude extract was reconstituted in distilled water and subsequently subjected to *n-*hexane partitioning, to produce the *n*-hexane fraction, which had a yield of 11.6%.

### Short Term Toxicity Experiment

The short-term toxicity study was assessed using Lorke’s method, a widely accepted technique for preliminary toxicity evaluation (Lorke, 1983). Swiss albino mice were fasted for 12 hours before the study and randomly allocated into categories (n = 3 per group) and given oral doses of the *n*-hexane fraction of (10, 100, and 1000) mg/kg respectively. They were observed for 72 hours for mortality and behavioral responses. As no deaths were recorded at these doses, the next level dose regimen of **(**1600, 2400, and 5000) mg/kg respectively were administered to them. A control group received 10% Tween-20 in distilled water.

The median Lethal Dose (LD_50_) was calculated using the equation;


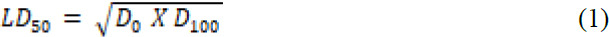


**D_0_** = Maximum dose at which all animals survived

**D_100_** = Minimum dose at which all animals died

### Repeated Dosing Toxicity Study

The repeated-dose toxicity of the test material was assessed following the OECD 407 guideline (OECD, 2008). Swiss albino rats were distributed at random into a category of five (n = 6 per group, both sexes) and fasted overnight before the treatment.

Group 1 (Control): 10% Tween-20 in distilled water (10 mL/kg)

Groups 2–5: 100, 200, 400, and 800 mg/kg of the *n*-hexane fraction (orally)

The extracts were given to the experimental animals through the oral route at 10 mL/kg body weight each day for a duration of 35 days. Throughout this period, the animals were closely monitored on a daily basis for signs of morbidity, distress, or mortality.

### Biochemical Assay

Upon conclusion of the treatments, blood specimens were obtained for investigation of key biochemical parameters which include; aspartate aminotransferase (AST), alanine aminotransferase (ALT), TRIG (triglycerides) and TC (total cholesterol). These biomarkers were measured using enzymatic colorimetric assay methods, as described by Huang *et al*. (2006). The findings are reported as; mean ± standard error of mean (SEM).

### Statistical Analysis

Data were analyzed using GraphPad Prism 8.0. Differences among groups were analyzed using one-way ANOVA, followed by Tukey’s post hoc test (Abdi and Williams, 2010). A p-value of less than 0.05 was considered statistically significant.

### Histological Analysis

The liver, kidney, lung, and spleen were carefully removed and immediately placed in formalin (10%) to preserve cellular integrity. Tissue sections, measuring 3–5 mm in thickness, were processed using the paraffin embedding technique and thereafter treated with hematoxylin and eosin (H&E) stain, following the method outlined by Bancroft and Gamble (2008). Histopathological alterations were assessed under a light microscope, and photomicrographs were examined at 100× magnification.

## Results

### Acute toxicity study of *Uvaria chamae* leaf n-hexane fraction

The acute toxicity results via the oral route in mice is shown in table 1. No death occured at the maximum dose and the LD_50_ value was estimated as > 5000mg/kg body weight.

**Table 1:** Mortality Effects of *Uvaria chamae* Leaf *n*-Hexane Fraction in Acute Toxicity Study on Mice

Time (hr)	Control	Doses (mg/kg)					

		10	100	1000	1600	2900	5000
24	0/3	0/3	0/3	0/3	0/3	0/3	0/3
48	0/3	0/3	0/3	0/3	0/3	0/3	0/3
72	0/3	0/3	0/3	0/3	0/3	0/3	0/3

The data presented in table 2 shows the effect of the *n*-hexane leaf fraction of *U. chamae* at dosage levels; 100, 200, 400, and 800 mg/kg on the liver biochemical parameters of the treated test animals and the standard group.

**Table 2:** Effect of *n*-hexane fraction of *U. chamae* on biochemical parameters in the liver

GROUP	AST(U/L)	ALT(U/L)	TC(mmol/L)	TRIG(mmol/L)
CONTROL	4802.82±345.29	6060.88±181.28	2.37±0.36	2.18±0.35
100 (mg/kg)	4953.61±234.93	5977.76±105.10	2.76±0.58	4.53±0.51*
200 (mg/kg)	4744.28±471.80*	5151.96±202.10	2.19±0.57	3.04±0.36
400 (mg/kg)	3457.12±217.34*	5130.05±190.91*	2.59±0.37	2.88±0.21
800 (mg/kg)	5382.37±270.42*	4041.83±273.89	2.40±0.17	1.65±0.17

Data are presented as Mean±SEM (n=6). The asterisks (*) indicates statistical significance (P.0.05) relative to the control

In the 400 mg/kg treatment group, AST levels showed a significant reduction, whereas a notable increase was observed at the 800 mg/kg dosage compared to the control. A significant elevation in ALT levels was detected only at the 400 mg/kg dose. Meanwhile, TC levels remained relatively unchanged across all treatment groups, with no statistically significant variations.

The TRIG levels significantly increased at a dosage of 100 mg/kg; however, the 800 mg/kg treatment group exhibited the lowest TRIG concentration, indicating potential modulation of lipid metabolism at higher doses. This data suggests a dose-specific effect of *U. chamae n*-hexane leaf extract on liver enzymes of the experimental animals.

Table 3 presents data on the effects of *U. chamae n*-hexane leaf extract at doses of 100, 200, 400, and 800 mg/kg on serum biochemical parameters in the experimental animals compared to the control group.

**Table 3:** Effect of *n*-hexane fraction of *U. chamae* on the biochemical parameters in the serum

GROUP	AST(U/L)	ALT(U/L)	TC (mmol/L)	TRIG (mmol/L)
CONTROL	150.69±5.83	45.62±4.60	2.32±0.22	1.34±0.05
100 (mg/kg)	145.33±4.58	111.29±6.80*	3.14±0.23*	1.81±0.06*
200 (mg/kg)	269.83±49.51*	167.32±18.57*	2.65±0.21	1.67±0.07*
400 (mg/kg)	360.61±20.26*	130.13±7.22*	3.24±0.15*	1.65±0.10*
800 (mg/kg)	307.44±22.70*	150.87±9.38*	2.96±0.17	1.59±0.06

Values are expressed as Mean± SEM of 6 animals per group. The * values show significant (P.0.05) difference from the control at 95% confidence level

The 200, 400, and 800 mg/kg treatment groups showed a significant increase in AST levels, while ALT levels significantly increased across all treatment groups compared to the control. TC levels also rose in all treatment groups, with the 200 and 400 mg/kg doses showing statistically significant differences. Similarly, TRIG was elevated in all treatment groups, with significant increases observed at 100, 200, and 400 mg/kg. These results suggest a dose-specific effect of *U. chamae n*-hexane leaf extract on serum enzymes in the experimental animals.

### Histopathological studies

**Plate 1:**
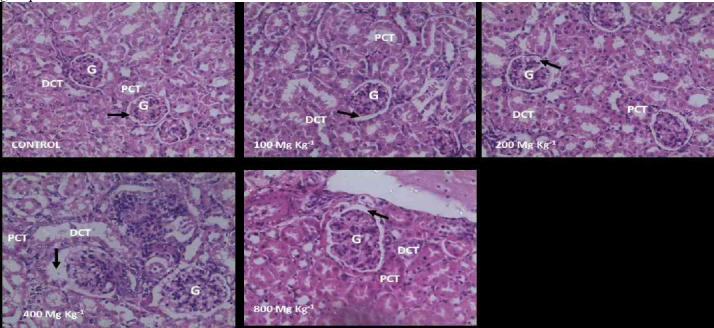
Histological examination of rat kidney sections (H&E stain, 400x magnification) The tissue cellular structure of the Bowman Space (arrow) of the glomerulus (G), proximal convoluted tubules (PCT) and distal convoluted tubules (DCT) of the kidney showed no distortion for control, 100 mg kg^-1^ and 200 mg kg^-1^ treated groups. Enlargement of Bowman space, interstitial edema, necrosis and extraglomerular hypercellularity were observed in groups given higher doses of the extract.

**Plate 2:**
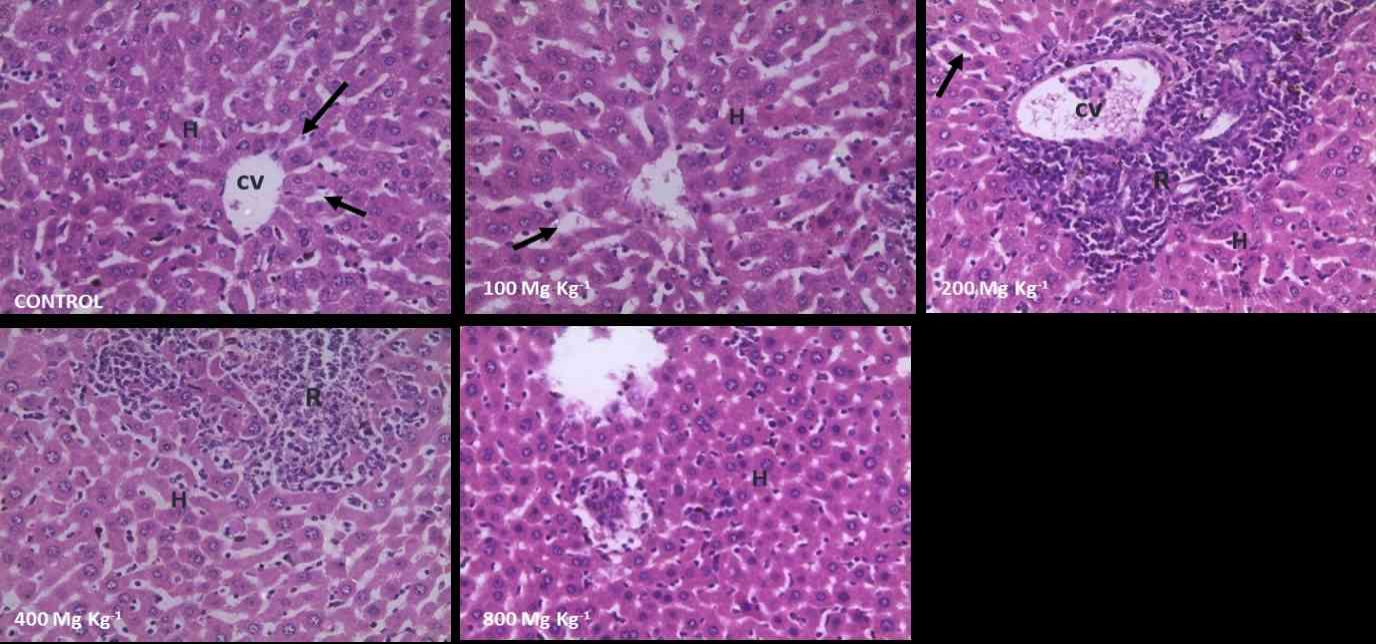
Histological examination of rat liver sections (H&E stain, 400x magnification) The liver structures of both the control and the treated groups are presented (Plate 2). Across all groups, hepatocytes (H) are organized in plates extending outward from the central vein (CV), with sinusoids (indicated by arrows) interspersed between these plates. The liver architecture remained intact in both the control group and the group administered 100 mg/kg of the extract. In these groups, hepatocyte plates were well-structured, sinusoidal spaces were distinct, and the central veins appeared normal. However, in groups treated with higher doses of the extract, distortion of the normal liver architecture and extrahepatic hypercellularity (R) were observed indicating an increased number of cells outside the normal hepatic tissue. These changes suggest that while lower doses of the extract (100 mg kg^-1^) did not significantly affect liver structure, higher doses led to noticeable alterations in liver morphology.

**Plate 3:**
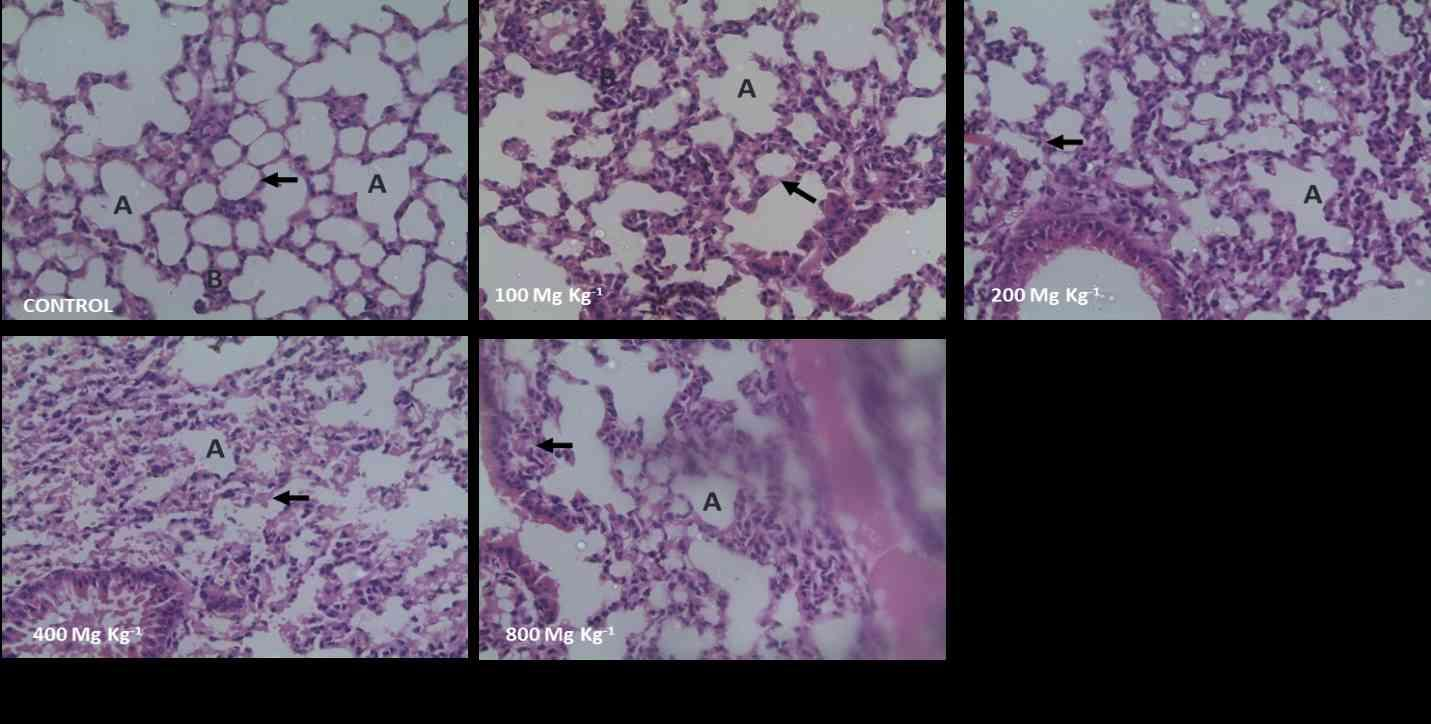
Histological examination of rat lung sections (H&E stain, 100x magnification) The lung structure appearance in both control groups and rats treated with the extract are presented. In the control group, the lungs displayed a normal spongy appearance, characterized by numerous air-filled alveolar sacs (A) connected to respiratory bronchioles (B). The alveolar walls (indicated by arrows) in the control group were thin, facilitating efficient gas exchange. Conversely, rats treated with the extract exhibited dose-dependent alterations in their lung tissue. Notably, all treated groups except the control, showed thickened alveolar walls and enlarged interalveolar septa, indicating pulmonary emphysema in the functional respiratory regions.

**Plate 4:**
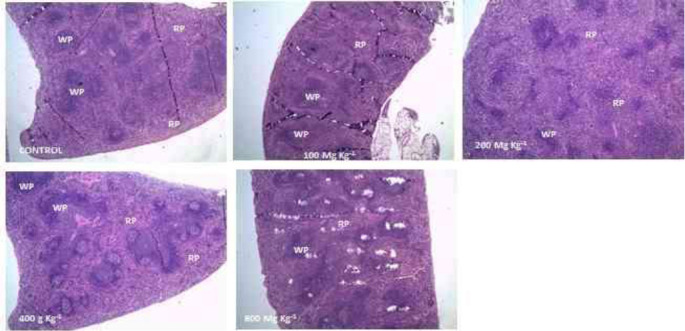
Histological examination of rat spleen sections (H&E stain, 40x magnification) The Photomicrographs of the spleen sections from the experimental rats as presented in plate 4 revealed two distinct regions: the white pulp (WP) containing lymphoid nodules, and the red pulp (RP). While the control group (untreated rats) showed a normal distribution and concentration of lymphoid follicles in the white pulp, there was a noticeable decrease in the concentration of lymphoid follicles within the white pulp of the extract treated groups in a dose dependent manner. The main histological change identified during this study was lymphoid depletion

The histopathological evaluations are presented in Plates 1 to 4, illustrating the effects of *Uvaria chamae* *n*-hexane leaf fraction at different doses on the kidney, lung, liver, and spleen of the treated test animals compared to the control group.

## Discussion

### Acute toxicity studies of *Uvaria chamae* leaf n-hexane fraction

The *n*-hexane fraction of *U. chamae* at all the doses deployed did not cause any mortality in mice within 72 hours after administration, indicating that the LD_50_ value is greater than 5000 mg/kg body weight. This suggests that this fraction is non-toxic in acute doses and may be considered safe for animal administration. These findings are corroborated in literature where an acute toxicity level of 7,080mg/kg with the aqueous-ethanol extract of *U. chamae* has been reported (Emordi *et al.*, 2015). According to OECD (2008) test guidelines, substances with LD_50_ values greater than 5000 mg/kg are classified as non-toxic. This supports the safe use of *U. chamae* for ethnomedicinal purposes, particularly regarding acute dose administration.

### Sub-chronic toxicity study of *Uvaria chamae* leaf *n*-hexane fraction

### Biochemical estimations of *U. chamae* leaf *n*-hexane fraction in the liver

A dose-dependent and significant reduction in the level of alanine amino transferase (ALT) liver enzyme marker was observed in rats treated with the *Uvaria chamae* leaf n-hexane fraction, while the aspartate amino transferase (AST) liver enzyme marker was significantly lower compared with the control only at the 400 mg/kg dose. High levels of transaminases are typically released into the bloodstream when liver cells experience stress, and therefore, increased AST levels are generally associated with liver damage or dysfunction (Pratt and Kaplan, 2000). Consequently, the decrease in ALT levels across all treatment doses, along with the lower AST level observed at 400 mg/kg, suggests a dose-dependent and dose-specific hepatoprotective effect of the extract. Conversely, in the 800 mg/kg treatment group, there was an increase in AST levels, indicating damage or stress in the liver at higher doses of *U. chamae*. In the context of these findings, rats treated with the ethanol root extract of *Uvaria chamae* at doses below 500 mg/kg exhibited low AST and ALT liver enzyme levels (Olumese *et al.*, 2018); however, at doses above 500 mg/kg, these enzyme levels increased significantly.

There are no significant changes in total cholesterol (TC) levels of the liver across the treatment doses when compared to the control, thus suggesting that the *n*-hexane fraction of *U. chamae* leaf does not have any notable effect on cholesterol levels of the experimental animals. Conversely, a dose-dependent effect was observed in the triglycerides (TRIG) levels of the rats, with an initial spike at lower dose (100 mg/kg), followed by a gradual reduction, eventually resulting in significantly lower levels at 800 mg/kg. Obisike *et al*. (2016) also reported that the aqueous extract of *Allium sativum* showed lower triglyceride (TRIG) lowering activity in rats at a higher concentration of 1000 mg/kg, while the 100 mg/kg treatment group exhibited higher TRIG levels. This fluctuation suggests that lower doses may cause disruption in TRIG metabolism, while the higher doses might exert a regulatory effect, which could be a form of feedback regulation potentially indicating a beneficial effect on lipid regulation at higher concentrations. Likewise, another study reported that Red Yeast Rice (RYR), a monacolin K-rich natural extract, exhibited a similar increase in triglyceride levels in treated rats at a lower dose of 1 mg/kg compared to the 3 mg/kg treatment (Mollace *et al.*, 2022). The phenomenon whereby lower concentrations of a drug demonstrated limited, no effect, or even worsened the condition before reaching the specific threshold for beneficial effects is referred to as the hormesis effect (Mattson and Calabrese, 2010).

### Biochemical estimations of *Uvaria chamae* leaf n-hexane fraction in the serum

There was a notable increase in serum ALT levels compared to the control. Additionally, serum AST levels exhibited a significant rise at higher doses. In the administration of the aqueous fraction of an aqueous-alcoholic extract of *Hibiscus sabdariffa* L, it has equally been reported that serum ALT and AST levels were significantly increased in all the treatment groups of albino rats (Akindahunsi and Olaleye, 2003). Likewise, Saeed *et al*. (2008) reported that there was an increase in the AST and ALT levels of experimental rats treated with the aqueous extract, ethyl acetate fraction, and butanol fraction of *Cephalotaxus sinensis*. Additionally, both TC and TRIG concentrations showed an increase compared to the control. The total cholesterol and triglyceride serum concentration were similarly increased compared to the positive control (untreated rats) in a cognate study (Banda *et al.*, 2018).

When the leaves of *Lannea edulis* crude aqueous extracts were administered to rats, there was an increase in the total cholesterol of rats treated with *Nauclea latifolia* leaf methanol fraction (Effiong *et al.*, 2014). The elevation of ALT and AST biochemical parameters in the serum of rats treated with *Uvaria chamae* root ethanol extract when compared with the control has been document in publications (Olumese *et al.*, 2018). The findings reveal that while ALT and AST levels decreased in hepatic tissues at certain doses, their corresponding increase in serum, along with elevated total cholesterol (TC) and triglyceride (TRIG) levels, suggests potential hepatocellular leakage. In contrast, TC levels in the liver remained stable.

This pattern indicates that although *Uvaria chamae* *n*-hexane fraction may exhibit hepatoprotective effects at lower doses, it could also pose a risk of systemic toxicity with prolonged use or higher doses. The opposing trends between liver and serum parameters suggest a complex mechanism of action, potentially affecting hepatic metabolism and lipid homeostasis.

Given the liver’s central role in detoxification, metabolism, and energy regulation (Selden and Hodgson, 2004; Hall and Hall, 2020), its biotransformation processes may explain why the *n*-hexane fraction had only moderate effects on liver enzyme markers while causing significant elevations in serum biomarkers in this study.

Certain herbal remedies, even when taken within therapeutic doses, and especially when taken in overdose, have been linked to hepatotoxicity. Over 900 medicinal compounds have been associated with liver toxicity, making hepatotoxicity one of the primary reasons for drug withdrawals from the market (Kaplowitz, 2021). These compounds often cause subclinical liver damage, typically detected through abnormal liver enzyme test results. Drug-induced liver injury has significant clinical impact, accounting for half of all acute liver failure cases and also contributing to approximately 5% of hospitalizations (Watkins and Woodcock, 2018).

AST and ALT are transaminase enzymes located within both the cytoplasm and mitochondria of various tissues, including the liver, erythrocytes, heart, skeletal muscles, kidneys, and pancreas (Vroon and Israeli, 1990). These enzymes serve as sensitive biomarkers, and their quantitative assessment is crucial in detecting hepatocellular damage (Mamary, 2002; Marra *et al.*, 2011). When damage occurs, these enzymes leak from the damaged tissues into the bloodstream, resulting in increased serum concentrations, thereby making them reliable indicators of tissue integrity and function. Elevated levels of these enzymes in the serum typically indicate tissue damage or cellular injury (Iweala *et al.*, 2019), thus justifying the observations in the reduction of the liver enzymes and lipid biomarkers compared to that of the serum as observed in this study.

### Histomorphology studies

The histopathological assessments of the kidney, liver, lungs, and spleen of the animals used in this study show various degrees of distortions, especially at the higher doses when compared with the control group, as shown in Plates 1 to 4. The enlargement of the Bowman’s space of the glomerulus, as observed in rats treated with higher doses of *Uvaria chamae* *n*-hexane extract, can indicate glomerular injury, possibly caused by pressure or damage to the glomerular membrane. The observed interstitial edema might be caused by the accumulation of fluid within the renal interstitium, which usually occurs as a result of inflammation or tissue injury, possibly due to the extract’s toxicity. The kidney’s cellular necrosis is also indicative of cytotoxic effects, while the observed extraglomerular hypercellularity might indicate an inflammatory response to the extract’s components. Similarly, the presence of interstitial vascular congestion, dilatation, and infiltration of lymphocytes can be detected in the kidney of rats treated with the aqueous extract of *Uvaria chamae* root (Olumese *et al.*, 2018).

At higher doses of *Uvaria chamae* n-hexane extract treatment, there were noticeable alterations, as well as extrahepatic hypercellularity, suggesting an increased number of cells outside the normal hepatic tissue. Mild vascular congestion with lymphocytic infiltration, suggesting inflammatory and circulatory changes in the liver of rats treated with *Uvaria chamae* aqueous root extract, has been reported (Olumese *et al.*, 2018). Additionally, an apparent lack of histopathological changes in the liver tissues studied was observed in another study (Ejeh *et al.*, 2019).

The liver histopathological response to a stress condition is usually characterized by significant cellular, structural, and metabolic changes. Hepatocyte cellular hypertrophy often manifests as enlarged cell sizes that deviate from normal dimensions. This transformation is usually accompanied by notable accumulations within the cytoplasm, including increased lipid droplets and glycogen deposits, which suggest disrupted metabolic processes. In response to these, there is an adaptive proliferation of cytoplasmic organelles, reflecting the liver’s effort to manage such metabolic stress or injury. These morphological changes are thus indicative of a deliberate cellular attempt at adaptation, suggesting an underlying hepatic pathological condition (Al-Jborrey and Al-Shahwany, 2017). Also, in this study, thickening of the alveolar walls and enlargement of the interalveolar septum, indicating pulmonary emphysema, were predominantly observed in the functional respiratory areas of the lungs in all treated rat groups. They also exhibited a dose-dependent effect, without any changes in the control group. The connective tissue architectural framework of the pulmonary connective tissue plays a major role in maintaining its structural stability for efficient gas exchange and respiratory dynamics (Adluri *et al.*, 2017). The observed emphysema in rats treated with the *Uvaria chamae* *n*-hexane fraction suggests a breakdown of the fibers of the connective tissue framework. This kind of degradation has been associated with exposure to certain toxic compounds, including those found in medicinal plants (Ghosh, 2020). Similarly, it has been reported that nicotine, through free radicals’ release, causes deterioration of the connective tissue framework, leading to lung damage in the form of emphysema in experimental rats treated with nicotine (Adluri *et al.*, 2017).

The main variation observed in the spleen histopathological examination of rats treated with the *n*-hexane fraction of *Uvaria chamae* was a dose-dependent reduction in lymphoid follicles in the treated groups, while the untreated rats maintained a normal distribution and concentration of lymphoid follicles. In contrast to the control group (untreated rats), which displayed a regular distribution and density of lymphoid follicles in the white pulp, the treated groups showed a noticeable decline in the concentration of these follicles in the white pulp, corresponding to the dose. The principal histological change identified in this study was lymphoid depletion. The spleen plays an essential role in the immune system, with two key regions: the red pulp, responsible for filtering blood and removing aged or damaged red blood cells, and the white pulp, which contains immune cells like T and B cells that are crucial for immune defense against infections (Mattson and Calabrese, 2010). In this study, a dose-dependent reduction in the density of lymphoid follicles within the white pulp was evident in the treated animals. Meanwhile, the control group exhibited a normal distribution and concentration of lymphoid follicles in the white pulp. A similar study reported a decrease in lymphoid white pulp concentration in spleen sections from animals treated with a 500 mg/kg body weight dose of the crude ethanolic stem extract of *Costus afer* (Obisike *et al.*, 2016). Another study reported slight lymphocyte hyperplasia in the spleens of experimental rats treated with the ethanol leaf extract of *Terminalia chebula* at all tested doses and attributed this condition to a heightened immune response (Ukpabi-Ugo *et al.*, 2023). In the course of this study, lymphocyte depletion or hypoplasia, that is, a reduction or loss of lymphocytes within the lymphoid tissues, was observed. This condition is typically indicative of a weakened immune response. This histological observation, along with other findings in this study, supports that the *Uvaria chamae* *n*-hexane fraction may have deleterious effects at sub-chronic doses. Therefore, caution should be exercised with its use.

## Conclusion

This study highlights the toxicity risks associated with prolonged use of *U. chamae* leaf *n*-hexane fraction**,** as extended exposure resulted in biochemical and histological alterations, showing potential hepatic and renal damage**.** Further research is needed to establish safe long-term dosage thresholds in both experimental models and clinical applications. Therefore, caution is recommended when using this plant for extended periods, and proper dosing should be carefully determined for its intended therapeutic use.

### Conflict of Interest Declaration

The authors declare that there is no conflict of interest in any form regarding the publication of this article.

**List of abbreviations used:**
U. chamae: *Uvaria chamae*;
AST: Aspartate Aminotransferase;
ALT: Alanine Aminotransferase;
TRIG: Triglycerides;
TC: Total Cholesterol;
LD_50_: Median Lethal Dose;
OECD: Organisation for Economic Co-operation and Development;
H&E: Hematoxylin and Eosin;
CV: Central Vein;
PCT: Proximal Convoluted Tubules;
DCT: Distal Convoluted Tubules;
SEM: Standard Error of Mean;
ANOVA: Analysis of Variance;
WP: White Pulp;
RP: Red Pulp;
NRC: National Research Council;
RYR: Red Yeast Rice
